# Prevalence and profile of isoniazid mono-resistant tuberculosis in Pakistan: Insights from a decade of surveillance

**DOI:** 10.12669/pjms.42.(ICON26).15695

**Published:** 2026-04

**Authors:** Nazia Khursheed, Syeda Mahnoor Zafar, Sunil Asif, Fareeha Adnan

**Affiliations:** 1 Nazia Khursheed, FCPS. Chair, Clinical Laboratory, Indus Hospital & Health Network, Korangi, Karachi, Pakistan; 2 Syeda Mahnoor Zafar, PharmD. Senior Research Scientist, ORIC, Indus Hospital & Health Network, Korangi, Karachi, Pakistan; 3 Sunil Asif, Assistant Manager, TB Laboratory, Indus Hospital & Health Network, Korangi, Karachi, Pakistan; 4 Fareeha Adnan, Consultant Microbiologist, Laboratory, Indus Hospital & Health Network, Korangi, Karachi, Pakistan

**Keywords:** Drug-resistance, Isoniazid Monoresistance, Isoniazid, Tuberculosis (TB), LMICs, Prevalence

## Abstract

**Background & Objective:**

Isoniazid monoresistance poses a growing concern in tuberculosis (TB) management, as it can compromise treatment outcomes and increase risk of multidrug-resistant TB. With limited data on its prevalence and characteristics in Pakistan, identifying its prevalence and factors associated is imperative. This study, therefore aimed to identify prevalence and resistance profile of isoniazid mono-resistance isolates and association of isoniazid mono-resistance with demographic, TB category and patient status.

**Methodology:**

This retrospective study analyzed all *Mycobacterium tuberculosis* complex (MTBC) culture-positive samples received at the Indus Hospital & Health Network (IHHN) Microbiology Laboratory between 2015 to 2023. Samples with no growth or growth of organisms other than MTBC, were excluded. Data were analyzed using SPSS version 26.

**Result:**

A total of 5,820 MTBC-positive cultures were evaluated. Among these, 140 (2.4%) were identified with isoniazid monoresistance. The prevalence fluctuated across the study period, with the highest rate observed in 2023 (3.5%). Most patients with isoniazid monoresistance had pulmonary TB (117; 83.6%) compared to extrapulmonary TB (23; 16.4%). The majority were newly enrolled cases (127; 90.7%). No statistically significant associations were found between isoniazid monoresistance and patient demographics or TB category.

**Conclusion:**

The prevalence of isoniazid monoresistance remained relatively low but showed a rising trend, peaking in 2023. Most cases occurred in newly enrolled pulmonary TB patients. Continuous surveillance and early detection are essential to guide treatment strategies and prevent the emergence of multidrug resistance.

## INTRODUCTION

Tuberculosis, despite the availability of antituberculosis treatments for over 50 years, continues to be the leading infectious cause of mortality worldwide.^1^ According to the WHO Global Tuberculosis Report of 2023, 81% of the deaths in 2022 were caused by tuberculosis (TB), making it the second most common cause of death.^2^ Isoniazid and rifampicin form the backbone of first-line tuberculosis treatment regimens due to their complementary actions. Isoniazid provides rapid bactericidal activity against actively replicating bacilli, while rifampicin exerts sterilizing effects on dormant populations, minimizing relapse risk.^3^ However, treatment for tuberculosis and infection control are made increasingly challenging by drug-resistant tuberculosis (DR-TB), a growing global problem.^4^ The rise in mortality reflects rising burden of undiagnosed and untreated TB, which highlights risk of drug-resistant tuberculosis; a serious threat to public health.

Among five DR-TB categories delineated in the WHO Global Tuberculosis Report, including isoniazid-resistant TB(Hr-TB); rifampicin-resistant TB (RR-TB); multidrug-resistant TB (MDR-TB, resistant to at least isoniazid and rifampicin); pre-extensively drug-resistant TB (pre-XDR-TB, resistant to rifampicin and any fluoroquinolone); and extensively drug-resistant TB (XDR-TB, resistance to rifampicin, any fluoroquinolone, and at least one additional Group A drug such as bedaquiline or linezolid.^2^ Isoniazid mono-resistant tuberculosis (strains resistant only to isoniazid), is of significant concern.^5^ Isoniazid works by inhibiting InhA, an enzyme in *Mycobacterium tuberculosis* that plays a central role in mycolic acid biosynthesis, essential for bacterial cell wall. Resistance to INH is caused by mutations in the *katG* or *ahpC* or *fabG1-inhA* operon’s promoter regions.^4^ Evidence indicates that inadequate management of undetected isoniazid mono-resistance can amplify the emergence of resistance to rifampicin, highlighting its global public health impact.^6^

Globally, prevalence of isoniazid mono-resistance ranges from 4% to 48% with isoniazid-resistant, rifampicin-susceptible TB estimated to affect approximately 11% of patients.^7^,^8^ In Pakistan, reported rates are 3% among new cases, 6.3% among previously treated cases, and 4.6% overall.^7^ The rates varied according to the site of infection, with higher resistance observed in pulmonary tuberculosis (PTB, 9.8%) compared to extrapulmonary tuberculosis (EPTB, 6.8%).^9^

Early and accurate diagnosis of drug-resistant tuberculosis is crucial for optimal treatment and disease prevention.^2^,^10^ Primary methods used in Pakistan for diagnosing TB include Xpert testing (Genotypic method) and culture and drug sensitivity testing (phenotypic DST method). Phenotypic DST is considered the gold standard for detecting drug-resistant M. tuberculosis since it may detect mutations at low levels (as low as 1%). However, a major limitation is the prolonged turnaround time required for phenotypic drug susceptibility testing (pDST)^10^ which made genotypic drug susceptibility testing by GeneXpert MTBC/RIF assay the screening method for tuberculosis and Rifampicin resistance. Resistance profile of other anti-TB drugs is underreported and treated as drug-susceptible TB.^5^,^11^,^12^

The aim of the study was to determine prevalence and resistance profile of Mycobacterium tuberculosis isolates exhibiting isoniazid monoresistance and explore its association with patient’s demographic characteristics, tuberculosis (TB) category, and treatment status over the past decade at Indus Hospital and Health Network (IHHN). The findings were intended to address the knowledge gap regarding isoniazid monoresistance, highlight the significance of Xpert- XDR as a screening tool promote further research on resistance patterns in Pakistan and the South Asian region, and support clinicians in optimizing treatment strategies.

## METHODOLOGY

This observational study was conducted at the Microbiology laboratory of IHHN in Karachi, Pakistan, using retrospective data from 2015-2023. The study population involve MTBC patients whose culture was done at IHHN. Non-probability consecutive sampling was used.

### Ethical Approval:

The study was approved by the Institutional Review Board (IRB) of IHHN (study number IHHN_IRB_2023_09_008; dated September 13, 2023).

### Inclusion Criteria:

- All samples positive for MTBC on culture done between 2015-2023, at IHHN Microbiology laboratory were included.

### Exclusion Criteria:

- Exclusion was done based on no bacterial growth or growth of bacteria other than MTBC.

### Data Collection Procedure:

Baseline characteristics, demographic and clinical data of study population were extracted from Hospital Management Information System (HMIS). Variables included patient’s age, gender, sample type, susceptibility pattern of TB isolates, and patient visit status.

### Microbiological Procedures:

Clinical specimens were processed according to World Health Organization (WHO) guidelines using the standard N-acetyl-L-cysteine–sodium hydroxide (NALC-NaOH) digestion and decontamination method. Smears were stained with auramine-O and examined for acid-fast bacilli. For isolation of *Mycobacterium tuberculosis* Complex (MTBC), both liquid medium Mycobacterium Growth Indicator Tube (MGIT; BACTEC MGIT 960 system, Becton Dickinson, USA) and solid Lowenstein-Jensen (LJ) medium were inoculated and incubated under standard conditions. Growth suggestive of MTB was confirmed using MPT64 antigen detection assay.

### Drug Susceptibility Tests and Quality Control:

The pulmonary and extrapulmonary *Mycobacterium tuberculosis* isolates were subjected to drug susceptibility testing (DST) using the BACTEC MGIT 960 system (Becton Dickinson, USA) as per manufacturer’s instructions and World Health Organization (WHO) guidelines.^13^ The final drug concentrations used were: streptomycin 1.0 μg/mL, isoniazid 0.1 μg/mL, rifampicin 1.0 μg/mL, ethambutol 5.0 μg/mL, pyrazinamide 100 μg/mL, kanamycin 2.5 μg/mL, capreomycin 2.5 μg/mL, amikacin 1.0 μg/mL, ofloxacin 2.0 μg/mL, moxifloxacin 1.0 μg/mL and 0.25 μg/mL, and levofloxacin 1.0 μg/mL. Isoniazid Critical concentration of 0.1 μg/mL was used according National tuberculosis control program guidelines (NTP).

Fluorescence-based Growth Units (GU) in drug-containing tubes were compared with those of drug-free growth control by the MGIT 960 software algorithm. When GU of the control tube reached 400 (within 4–13 days of incubation), GU values of corresponding drug-containing tubes were analyzed. Isolates were interpreted as *susceptible* if GU ≤ 100 and *resistant* if GU > 100. Isolates demonstrating growth in the control tube but no-growth in drug-containing tubes were considered susceptible; whereas growth in both indicated resistance.

### Quality Controls:

Quality control of each batch was performed with reference strain *Mycobacterium tuberculosis* H37Rv (ATCC 27294).

### Statistical Analysis:

The Data were analyzed using SPSS version 26. Normality of Quantitative variables such as age was assessed using Kolmogorov-Smirnov test and median (IQR) was reported. Categorical variables such as gender, TB categories (PTB or EPTB) and patient visit status were reported as frequencies and percentages. Chi-square/Fisher exact test was applied to identify significant association of age, gender, TB categories and patient visit status with occurrence of Isoniazid monoresistance. The age difference between Isoniazid monoresistance group was checked by using Mann-Whitney U test. P-values of 0.05 or less were considered statistically significant.

## RESULTS

### Demographic Distribution of MTB Patients:

The isoniazid monoresistance of 5,820 mycobacterium-positive cultures was examined. With 2,954 males (50.8%) and 2,866 females (49.2%), demographic distribution of all TB cases tested for isoniazid monoresistance in Table-I demonstrates near-equal gender representation. Median age of study population was 29 years (IQR 20-45), with an age range of 1 to 95 years (Table-I). Regarding TB category, majority of overall TB patients had PTB (n=5,033; 86.5%) compared to EPTB (n=787; 13.5%) (Table-I). Regarding visit status, most cases were newly diagnosed (n=4,965; 85.3%), followed by previously treated cases (n=807; 13.9%), cases with unknown treatment status (n=32; 0.5%), relapse cases (n=10; 0.2%), and cases under follow-up for treatment response (n=6; 0.1%) (Table-I).

**Table-I:** Demographic Distribution of overall TB patients tested for INH-Monoresistance.

***Gender***	
Male	2954(50.8)
Female	2866(49.2)
***Age***	
Median (IQR)	29(20-45)
Min-Max	(1-95)
***TB category***	
PTB	5033(86.5)
EPTB	787(13.5)
***Visit Status***	
Follow-up	06(0.1)
New enrolled	4965(85.3)
Previously Treated	807(13.9)
Relapse	10(0.2)
Unknown	32(0.5)
Total	5820(100)

### Prevalence and Trend of INH-Monoresistance:

**Fig.1:**
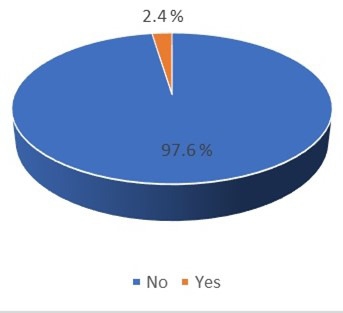
Proportion of isoniazid (INH) monoresistance among Mycobacterium tuberculosis (MTB)-confirmed patients, 2015–2023

**Fig.2:**
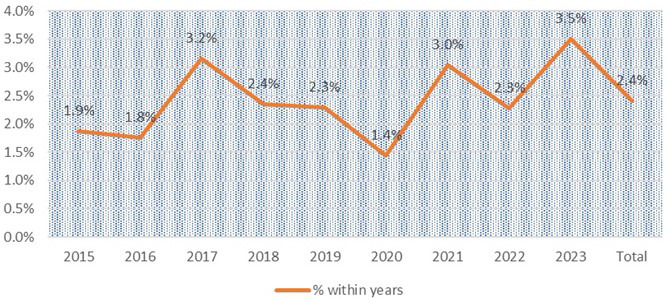
Trend of Isoniazid Mono resistance (INH) monoresistance among Mycobacterium tuberculosis isolates.

Of the total patients tested, 140 were identified with isoniazid mono-resistance, corresponding to a prevalence of 2.4% (Table-II, Fig.1). Prevalence of isoniazid mono-resistance varied across the years 2015–2023, with highest peaks recorded in 2017 (3.2%), 2021 (3.0%), and 2023 (3.5%), while lowest rates were observed in 2015 (1.9%), 2016 (1.8%), and 2020 (1.4%) (Fig.1 and 2).

**Table-II:** Association between patient demographics, TB category and IHN-monoresistance

Variables	Association with INH-Monoresistance			P value
	Yes	No	Total	
***Gender***				
Male	64 (45.7)	2890(50.9)	2954(50.8)	0.227^ꝉ^
Female	76(54.3)	2790(49.1)	2866(49.2)	
Total	140(100)	5680(100)	5820(100)
***TB category***				
PTB	117(83.6)	4916(86.5)	5033(86.5)	0.309^*ꝉ^
EPTB	23(16.4)	764(13.5)	787(13.5)	
Total	140(100)	5680(100)	5820(100)
***Age***				
Median (IOQ)	26(18-44)	29(20-45)	29(20-45)	0.129^ꝉ^
Min-Max	1-80	1-95	1-95
***Visit status***				
Follow up	-	06(0.1)	06(0.1)	0.298^ɫ^
New enrolled	127(90.7)	4838(85.2)	4965(85.3)	
Previously Treated	12(8.6)	795(14.0)	807(13.9)	
Relapse	-	10(0.2)	10 (0.2)	
Unknown	1(0.7)	31(0.5)	32(0.5)	
Total	140(100)	5680(100)	5820(100)	

* p<0.05, ꝉ Pearson Chi-square test Fischer exact test

### Association Of IHN- Monoresistance With Demographic Distribution, TB Category, and Patient Categories:

Demographic distribution of 76 (54.3%) females, and 64 (45.7%) males showed association with isoniazid monoresistance (Table-II). Median age of patients with isoniazid mono resistance was 26 years with a median range of 18 to 44 years. With respect to TB category, majority of patients with isoniazid mono resistance had PTB 117(83.6%) compared to EPTB 23(16.4). Majority of the study population who possess isoniazid mono resistance were newly enrolled (127 90.7%) compared to those previously treated (12; 8.6%). No statistically significant association was noted between isoniazid monoresistance and gender, age TB category or patients’ visit status (Table-II).

## DISCUSSION

Findings of the study provide pivotal insight into patterns of isoniazid monoresistance in context of Pakistani population; a country with a high burden of tuberculosis, ranking fifth on the global scale.^2^ Observed prevalence of isoniazid monoresistance, averaging 2.4% was predominantly detected among PTB samples (n=5,033; 86.5%); particularly noteworthy, given its peaks recorded in specific years 3.2% in 2017, 3.0% in 2021, and 3.5% in 2023. This was attributed to increase in number of collection points of IHHN in various areas of Sindh with high MDR TB burden from 2016 to 2019. This is akin to increased rise in a study of Saudia, which reported prevalence of 8.6% of isoniazid monoresistance between 2015 and 2019.^14^ Significantly higher prevalence was also observed in south African region with 21.7% of isoniazid monoresistance between 2011 and 2014.^15^

The onset of the COVID-19 pandemic curtailed TB sampling due to limited patient accessibility at hospital whereby shifting the sampling burden between 2021-2023 which potentially contributed to rise in isoniazid-resistant TB during 2021 and 2023. Fluctuations in resistance rates underscore a need for continuous surveillance and a comprehensive understanding of factors influencing resistance patterns.

Our findings indicate that a substantial proportion of patients with isoniazid monoresistance were newly diagnosed cases (127/140; 90.7%), while only a small fraction was previously treated (12/140; 8.6%). This pattern is consistent with findings which also reported that most isoniazid-resistant patients were newly diagnosed tuberculosis cases.^16^ Furthermore, amongst newly diagnosed MTB patients, single *inhA* mutations were more common compared to *katG* mutations in new cases as indicated by a study from South India, in which new cases with isoniazid resistance were more likely to have single *inhA* mutations (33.1%) compared to previously treated cases (20.9%).^17^

The fact that median age of 18–44 years is observed amongst isoniazid mono-resistant cases is a cause of concern. Rifampicin susceptibility often considered as isoniazid susceptible, resulting in prescribing of more powerful antibiotics at early age and or prescribing of already resistant drug contributes more to the MDR issue. This often-missed isoniazid monoresistance and interchangeably linked rifampicin resistance results in undermining of the real situation of isoniazid resistance. A similar finding is observed in another study by Micheni et.al which showed higher risk of isoniazid mono-resistance in patients of age 25 to 44 years in Southwestern Uganda.^18^ This calls for a paradigm shift in our approach for diagnosis using the comprehensive testing of XDR-Xpert and phenotypic drug testing. This strategy would improve identification of isoniazid monoresistance and allow more customized therapies to reduce future risk of drug resistance under supervised treatment.

Interestingly, the lack of association between isoniazid monoresistance and demographic variables such as gender, age, and TB category and patient’s visit status suggests that emergence of this resistance may not be influenced by conventional risk factors such as TB category and patient’s visit status, previously highlighted in studies of Micheni et al and MV Parsanna et al.^18^,^19^ but are similar to findings of studies that indicate no significant correlation between isoniazid monoresistance and demographic factors like gender and age,^19^ and that TB category does not appear to affect resistance patterns.^20^

Some studies also suggest that genetic factors and previous treatment history may influence resistance patterns, indicating a more complex interplay of factors in the emergence of isoniazid resistance.^21^,^22^ The absence of demographic correlations challenges prevailing narrative that certain populations are inherently more susceptible to developing drug-resistant TB. This finding is particularly significant as it indicates that broader epidemiological factors, possibly including socio-economic determinants, healthcare access, and treatment adherence, may play a more critical role in the emergence of isoniazid monoresistance.

Lastly, this study contributes valuable data on isoniazid monoresistance in Pakistan, emphasizing a need for surveillance and appropriate diagnostic strategies to address isoniazid monoresistance. It extrapolates that baseline isoniazid testing should be incorporated for bacteriologically confirmed TB, even in case of Rifampicin resistance. Future research should explore socio-economic and healthcare factors and evaluate targeted interventions. It calls for a multifaceted approach to TB management that prioritizes early detection and comprehensive treatment strategies, particularly among young adults who represent a critical demographic in the fight against tuberculosis. These are significant finding which needs to be addressed in Asian and African countries to prevent unwanted TB drugs resistance and promotion of appropriate treatment strategies.

### Strengths

Sample size of 5,820 which included pulmonary and extrapulmonary TB cases, a wide age range (1–95 years) and nearly equal gender representation, provided strong grounds for generalizability on prevalence of Monoisoniazid resistance. Furthermore, the use of culture-based laboratory confirmation ensures objective and reliable measurement of drug resistance.

### Limitations:

Detection of isoniazid monoresistance was based solely on phenotypic drug susceptibility testing (pDST), as genotypic methods were unavailable during study period. This warrants for more researches taking in account of clinical data, radiographic findings, and treatment outcomes, clinical correlation, and outcome assessment, in addition to variables used in this study.

### Author Contribution:

**NK**: Conceptualized the study, designed the methodology, written, reviewed original draft and also also supervised this study.

**SMZ:** Literature search and writing the introduction and discussion parts of manuscript.

**SA**: Contributed to data cleaning, methodology design, formal analysis, data visualization, and results interpretation.

**FA**: Questionnaire design, literature search and manuscript writing.

All authors have read the final version and are responsible and accountable for the accuracy and integrity of the work.
